# An Ancient Pathway Combining Carbon Dioxide Fixation with the Generation and Utilization of a Sodium Ion Gradient for ATP Synthesis

**DOI:** 10.1371/journal.pone.0033439

**Published:** 2012-03-29

**Authors:** Anja Poehlein, Silke Schmidt, Anne-Kristin Kaster, Meike Goenrich, John Vollmers, Andrea Thürmer, Johannes Bertsch, Kai Schuchmann, Birgit Voigt, Michael Hecker, Rolf Daniel, Rudolf K. Thauer, Gerhard Gottschalk, Volker Müller

**Affiliations:** 1 Göttingen Genomics Laboratory, Institute for Microbiology and Genetics, Georg August University, Göttingen, Germany; 2 Molecular Microbiology and Bioenergetics, Institute of Molecular Biosciences, Johann Wolfgang Goethe University, Frankfurt, Germany; 3 Max Planck Institute for Terrestrial Microbiology, Marburg, Germany; 4 Institute for Microbiology, Ernst Moritz Arndt University Greifswald, Greifswald, Germany; University of Groningen, The Netherlands

## Abstract

Synthesis of acetate from carbon dioxide and molecular hydrogen is considered to be the first carbon assimilation pathway on earth. It combines carbon dioxide fixation into acetyl-CoA with the production of ATP *via* an energized cell membrane. How the pathway is coupled with the net synthesis of ATP has been an enigma. The anaerobic, acetogenic bacterium *Acetobacterium woodii* uses an ancient version of this pathway without cytochromes and quinones. It generates a sodium ion potential across the cell membrane by the sodium-motive ferredoxin:NAD oxidoreductase (Rnf). The genome sequence of *A. woodii* solves the enigma: it uncovers Rnf as the only ion-motive enzyme coupled to the pathway and unravels a metabolism designed to produce reduced ferredoxin and overcome energetic barriers by virtue of electron-bifurcating, soluble enzymes.

## Introduction

**Figure 1:**
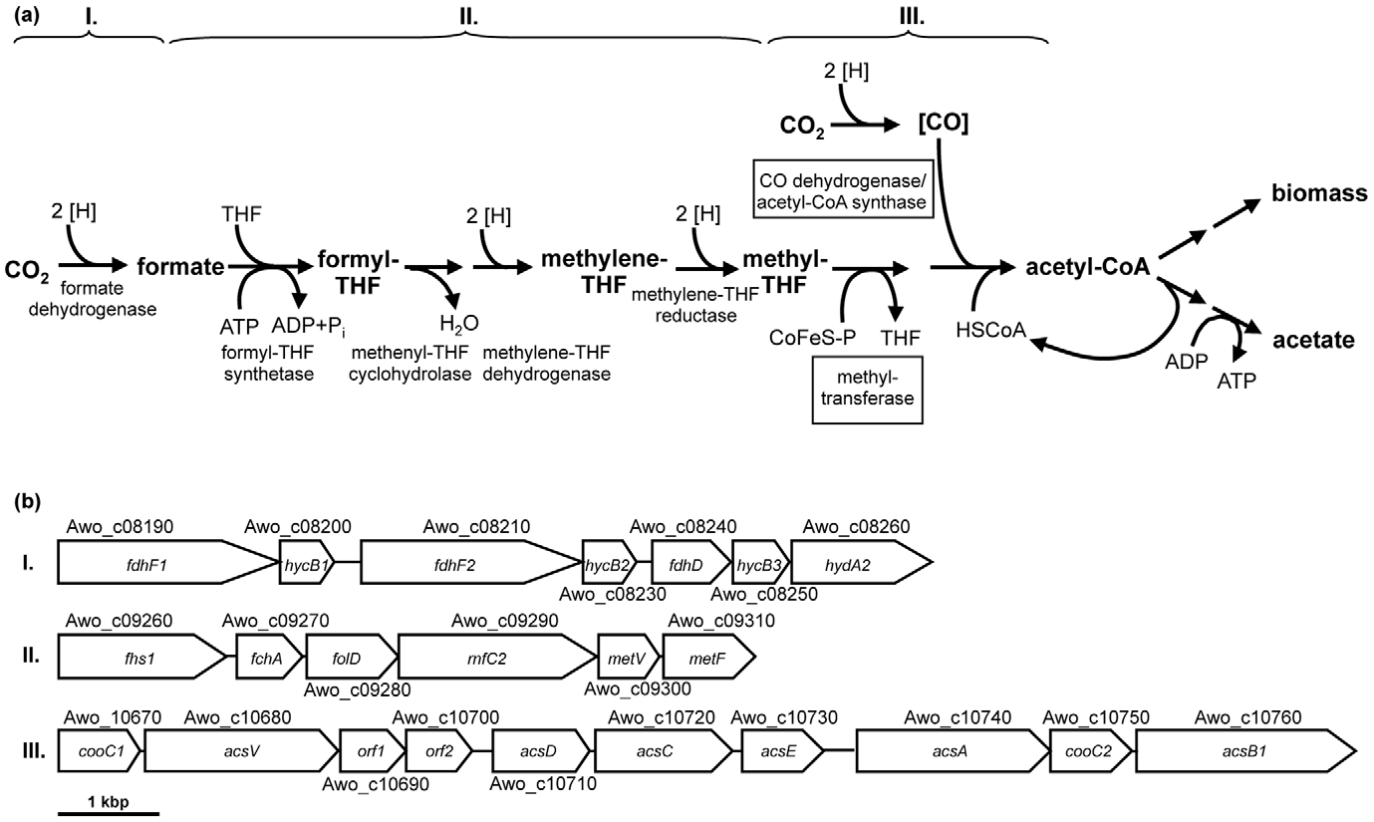
The enzymes of the Wood-Ljungdahl pathway (a) and the corresponding gene cluster (b). Cluster I: two genes for formate dehydrogenase (*fdhF*) are organized together with three copies of a FeS-containing subunit of a [FeFe]-hydrogenase (*hycB*), a formate dehydrogenase accessory protein (*fdhD*) and another subunit of the [FeFe]-hydrogenase (*hydA2*). Cluster II: genes for formyl-THF synthetase (*fhs1*), methenyl-THF cyclohydrolase (*fchA*), methylene-THF dehydrogenase (*folD*) and methylene-THF reductase (*metF, metV*) are organized together with a RnfC-similar protein (*rnfC2*). Cluster III: genes for the subunits of the CO dehydrogenase/acetyl CoA synthase complex consisting of CO dehydrogenase (*acsA*), acetyl-CoA synthase (*acsB1*), corrinoid-iron sulfur protein (*acsCD*) and methyltransferase (*acsE*) are organized together with two copies of a CODH nickel-insertion accessory protein (*cooC*), a corrinoid activation/regeneration protein (*acsV*) and two hypothetical proteins (Orf1 and Orf2).

The atmosphere in the early days of our planet was highly reducing and did not contain oxygen but gases such as molecular hydrogen and carbon dioxide. How life evolved under these conditions is a matter of debate but some researchers favour a pathway that combines two essential features: carbon fixation into organic molecules and, at the same time, generation of ATP [1]. The Wood-Ljungdahl pathway of carbon dioxide fixation is such a pathway and, therefore, could be considered to have evolved on earth very early [2], [3]. It is present in strictly anaerobic sulfate reducing bacteria and archaea, in methanogenic archaea and acetogenic bacteria [4]. Only in the latter it fulfills the dual function mentioned which is growth on molecular hydrogen and carbon dioxide according to:
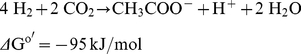
(1)The pathway involves reduction of carbon dioxide to formate, binding of formate to the cofactor tetrahydrofolate (THF) and subsequent reduction of the formyl group to methyl tetrahydrofolate. Another mol of carbon dioxide is reduced to enzyme-trapped carbon monoxide that is then condensed with a methyl group and coenzyme A to yield acetyl- CoA (Fig. 1). In the anabolic route, acetyl-CoA is the precursor molecule for biosynthetic reactions and in the catabolic route the precursor of acetate [5], [6], [7]. Acetyl-CoA allows for the synthesis of one mol ATP via substrate level phosphorylation. However, this ATP is required for formate activation to formyltetrahydrofolate leaving no ATP. Therefore, acetogenesis from 4 H_2_+2 CO_2_ must yield additional ATP by a mechanism other than substrate level phosphorylation. How this is achieved is unknown. The amount of energy gained from equation 1 would theoretically allow the generation of only 1.5 mol of ATP per mol acetate produced [8]. This calculation is based on a partial pressure of molecular hydrogen of 1 bar. However, at the low hydrogen partial pressures observed in the environment in which the acetogenic bacteria thrive the free energy change associated with reaction 1 is much more positive and sufficient to generate only a fraction of an ATP [9]. Therefore, acetogens clearly live at the thermodynamic limit of life.

Acetogens can be divided in two groups with respect to their energy metabolism [10]. One group contains cytochromes and quinones that are considered to be involved in a membrane-bound electron transport process that generates a proton potential across the membrane; they use a proton potential-driven ATP synthase. This can be considered as the evolutionary more advanced version of the pathway since it required the evolution of cytochromes [11]. The second group, to which the Gram positive bacterium *Acetobacterium woodii* belongs, represents a more ancient version of the pathway. It does not involve cytochromes and quinones but at least one ion pump, a sodium ion-translocating ferredoxin:NAD^+^ oxidoreductase (Rnf) [12]. The Rnf complex is a membrane-bound electron transfer complex containing iron-sulfur centers and flavins. Exergonic electron transfer from reduced ferredoxin (Fd^2−^) to NAD^+^ is found to be coupled with vectorial, electrogenic Na^+^ transport in inverted membrane vesicles of *A. woodii* [13]. Immunological studies revealed that the Rnf complex is present in cells grown on H_2_+CO_2_, underlining its importance in the bioenergetics of the Wood-Ljungdahl pathway [14]. The complex is also present in cells grown on formate, methanol, betaine or fructose and its synthesis was also independent of the electron acceptor used and, therefore, is constitutive [14]. The sodium ion potential across the membrane drives the synthesis of ATP *via* a membrane-integral Na^+^ F_1_F_O_ ATP synthase [15], [16].

Another argument that this version of the pathway has evolved early is that it uses Na^+^ as coupling ion. Membranes are more tight for Na^+^ than for H^+^ [17] which is essential for organisms living in ecosystems that have a high concentration of organic acids that function as “proton ferries” allowing protons to backenter the cell and thus destroying part of the already very little power available to fuel the ATP synthase. Therefore, sodium bioenergetics using a ΔpNa is considered an early step in the evolution of cellular bioenergetics [18].

*A. woodii* employs the Wood-Ljungdahl pathway without cytochromes and is one of the very few organisms known to completely rely on a sodium ion potential for energetic reactions [10]. It grows well in the absence of a proton potential across its membrane, and apparently uses a primary Na^+^ potential to fuel ATP synthesis, transport processes and flagellar rotation [16]. Thus it is a prime candidate to unravel the bioenergetics of what may have been one of the first pathways in coupling exergonic and endergonic reactions in life. Therefore, we sequenced the entire genome of *A. woodii* and performed a few key experiments to fill the gaps in understanding how the pathway is coupled to the synthesis of ATP.

## Results and Discussion

### Genetic organization and predicted properties of enzymes of the Wood-Ljungdahl pathway

General genome features of *A. woodii* are listed in Table 1. The genome of *A. woodii* consists of a single circular 4,044,785 bp chromosome with a GC content of 39.3%. 3473 protein-encoding ORFs, 5 rRNA clusters and 60 tRNA genes account for 85.1% of the genomic DNA. 71.1% of the predicted ORFs are with assigned functions (NCBI database, accession number CP002987). The genes encoding proteins of the Wood-Ljungdahl pathway are mainly found in three clusters on the chromosome (Fig. 1).

**Table 1:** General features of the *A. woodii* genome.

	*A. woodii*
Genome size (Mbp)	4.044785
protein encoding orfs	3472
percent coding (%)	85.1
G+C content (mol%)	39.34
rRNA cluster	5
tRNA	59
orfs with assigned functions	2469
orfs without assigned functions	1004

In cluster I, two genes, each encoding a formate dehydrogenase (*fdhF*), are present. The formate dehydrogenase catalyzes the first step in the pathway, the conversion of CO_2_ to formate. FdhF1 and FdhF2 are 79% identical and FdhF2 seems to be a selenocystein-containing protein. Apparently, it is the only selenocystein-containing protein encoded on the genome of *A. woodii*. The genes encoding selenocystein biosynthesis proteins are next to the cluster upstream of *fdhF1*. Furthermore, there are genes present that potentially encode a formate dehydrogenase accessory protein (*fdhD*), three copies of a putative FeS-containing electron transfer protein (*hycB*) and a subunit *(hydA2)* harbouring the active site characteristic of [FeFe]-hydrogenase. HycB1 and HycB2 are 82% identical and the encoding genes are downstream of *fdhF1* and *fdhF2*, respectively. Therefore, we suggest that HycB1 and HycB2 are electron transfer proteins specific for FdhF1 and FdhF2, respectively. HycB3 is only 34 and 33% identical to HycB1 and HycB2, respectively, and its localization in the gene cluster indicates that it is the electron output module for the hydrogenase HydA2.

Cluster II encodes the enzymes catalyzing the reduction of the formyl-group to the methyl-group, the formyl-tetrahydrofolate (THF) synthetase, methenyl-THF cyclohydrolase, methylene-THF dehydrogenase and methylene-THF reductase. The genetic organization is different from *Moorella thermoacetica* where these genes are spread all over the genome [19]. A second formyl-THF synthetase is found in *A. woodii* (Awo_c08040), whereas in *M. thermoacetica* and *Clostridium ljungdahlii* only one such enzyme is encoded. Interestingly, *metF*, the gene encoding the methylene-THF reductase is preceded in *A. woodii* by two genes (Awo_c09290, Awo_c09300) of unknown function. Homologues of Awo_c09300 are also found together with *metF* in many acetogens such as *M. thermoacetica* and *C. ljungdahlii*, but also in other bacteria and archaea like *Alkaliphilus metalliredigens, Geobacter sulfurreducens* or *Methanosarcina acetivorans*. In contrast, homologues of Awo_c09290 are only found in this region in *Ruminococcus obeum*, *Blautia hydrogenotrophica and Bryantella formatexigens*, and three members of the genus *Clostridium*. The possible function of these proteins is discussed below.

Acetyl-CoA is formed from methyl-THF and a second CO_2_ by the CO dehydrogenase/acetyl-CoA synthase complex. Genes coding for the subunits (*acsAB*) are found in cluster III (Awo_c10670-Awo_c10760) together with the genes coding for the corrinoid-iron sulfur protein (*acsCD*) and the methyltransferase (*acsE*). Additionally there are two genes in the cluster encoding for a CODH nickel-insertion accessory protein (*cooC*) and a corrinoid activation/regeneration protein (*acsV*) (Fig. 1.). The same genes are also clustered in *M. thermoacetica*, *Acethalobium arabaticum* and *C. ljungdahlii*. The genes for the phosphotransacetylase (Awo_c19620) and the acetate kinase (Awo_c21260) are located in other regions of the genome.

### The Rnf complex is the only membrane-bound electron transfer system of the Wood-Ljungdahl pathway

Inspection of the genome sequence ruled out that any of the orfs encoding for enzymes involved in carbon flow in the Wood-Ljungdahl pathway are membrane-bound and potential Na^+^ pumps. This was a surprise since it was speculated for decades that the methyl-THF:corrinoid-iron sulfur protein methyltransferase is a membrane integral, Na^+^-translocating enzyme [10]. This assumption was based on the finding of membrane-bound corrinoids in *A. woodii* [20] and the finding of a corrinoid-containing, Na^+^-translocating methyltransferase in methanogens [21]. *A. woodii* encodes 30 different methyltransferase systems involved in channelling methyl groups from various substrates into the central pathway, but none is predicted to be membrane-bound. The corrinoid-containing proteins found in the membrane fraction of *A. woodii* [20] may thus stem from “sticky” cytoplasmic methyltransferases.

Thus, the genome sequence revealed that Rnf is probably the only ion-pumping enzyme active during litho-autotrophic growth of *A. woodii*. The use of Rnf as the only coupling site in the pathway leads to important consequences. The E_0_′ of the Fd_ox_/Fd^2−^ couple is probably −500 mV and that of the NAD^+^/NADH couple is −320 mV (the E_0_′ of the Fd_ox_/Fd^2−^ couple is assumed to be near −500 mV because in *A. woodii* reduced ferredoxin is used as electron donor for the reduction of CO_2_ to CO, the E_0_′ of the CO_2_/CO couple being −520 mV [20]). The higher the Fd^2−^/NAD^+^ concentration ratio, the higher the energy that can be used for building up a sodium ion gradient. Therefore, the entire catabolic metabolism has to be optimized to produce a maximal Fd^2−^/NAD^+^ ratio. A maximal number of oxidation steps should produce reduced ferredoxin whereas a maximal number of reduction steps should use NADH as electron donor. This leads to a metabolism focused on ferredoxin.

### Overcoming a first energy barrier in the electron pathway

**Figure 2:**
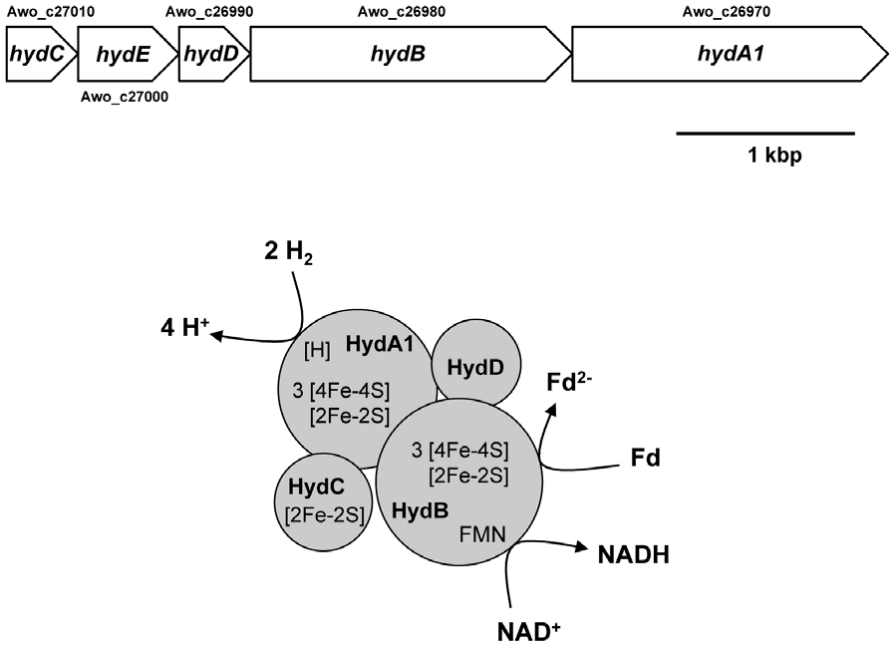
Genetic organization and subunit composition of the [FeFe]-hydrogenase of *A. woodii*. Awo_c27000 (HydE) is not a component of the active enzyme but may be involved in assembly or signalling.

In the first step of the electron transfer pathway, ferredoxin is reduced with H_2_ as reductant. This reaction is highly endergonic with a ΔG^o^′ = +18.5 kJ/mol (E_0_′ Fd_ox_/Fd_red_ = −500 mV; E_0_′ 2 H^+^/H_2_ = −414 mV) and thus requires the input of energy. One possibility to overcome this steep energy barrier is to drive the endergonic electron transfer from molecular hydrogen to ferredoxin by the electrochemical ion potential across the membrane through ion influx into the cell. Such a kind of reverse electron flow is catalyzed by Ech-type or related hydrogenases [22] but genes encoding such enzymes are absent from the chromosome. Moreover, the genome of *A. woodii* does not harbour genes encoding [NiFe]-hydrogenases but a putative operon consisting of 5 genes (*hydA1-hydE*, Awo_c26970-Awo_c27010, Fig. 2) that potentially encode a cytoplasmic multimeric [FeFe]-hydrogenase. *hydA1* encodes a putative catalytic hydrogenase subunit with a complete H-cluster, one [2Fe2S] and three [4Fe4S] clusters, based on sequence similarities. *hydB* codes for a putative iron sulfur protein with one [2Fe2S], three [4Fe4S] and a flavin binding site. *hydC* is the first gene of the cluster and codes for a protein with one predicted [2Fe2S] binding site. The protein encoded by *hydD* has apparently no cofactor binding sites. HydE has no similarity to hydrogenase subunits but is similar to ATP – binding domains of histidine kinases. Proteomic analysis of cells grown with H_2_+CO_2_ *vs.* cells grown on fructose revealed that the [FeFe]-hydrogenase is upregulated ∼3 fold during litho-autotrophic growth (Fig. S1, Table S1) which is consistent with the hypothesis that it catalyzes hydrogen activation.

The putative encoded multimeric hydrogenase (HydA-D) shares similarities with a trimeric hydrogen-evolving hydrogenase of *Thermotoga maritima* [23], [24] that consists of the three proteins HydA, HydB and HydC. Production of hydrogen from NADH+H^+^ is thermodynamically highly unfavourable and Schut and Adams [23] proposed a flavin-based electron bifurcating mechanism to overcome the energetic barrier. Flavin-based electron bifurcation was first shown in anaerobic clostridia as a new coupling mechanism in bioenergetics: the energy liberated during energetic “downhill” transfer of one electron from a donor to an acceptor is used to transfer another electron “uphill” to an acceptor with a more negative redox potential. That was first experimentally proven for the exergonic NADH+H^+^-driven reduction of crotonyl-CoA that is coupled with endergonic ferredoxin reduction [25], [26]. For the hydrogen-evolving hydrogenase of *T. maritima*, it is suggested that the energetic “downhill” transfer of two times one electron from ferredoxin (E_0_′ approx. –500 mV) to two protons (2 H^+^/H_2_; E_0_′-414 mV) drives the energetic “uphill” transport of two times one electron from NADH+H^+^ (E_0_′ = −320 mV) to two protons. In sum, the reaction is:
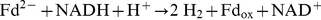
(2)Due to the similarity of the enzymes from *A. woodii* and *T. maritima* we suggest that the [FeFe]-hydrogenase of *A. woodii* catalyzes a reversal of equation 2, namely the reduction of one ferredoxin and one NAD^+^ with two H_2_ (Fig. 2). The finding that cell free extracts of *A. woodii* grown on fructose catalyze hydrogen-dependent NAD^+^ reduction only after the addition of FMN and ferredoxin purified from *Clostridium pasteurianum* is consistent with this hypothesis.

### Overcoming a first energy barrier in the carbon pathway

The first step in CO_2_ reduction is an endergonic reaction assuming that electrons are passed from molecular hydrogen to CO_2_ *via* NADH+H^+^ as a reductant (the standard redox potential of the CO_2_/formate couple is −430 mV). Electrons may be delivered *via* NADPH generated from NADH+H^+^ in an energy-dependent reaction driven by either a membrane-integral transhydrogenase [27] or an electron bifurcating soluble transhydrogenase [28]. However, homologues of these enzymes are apparently not encoded in *A. woodii*. But how are the electrons transferred from molecular hydrogen to carbon dioxide? As mentioned above, the formate dehydrogenase gene cluster harbors one gene (Awo_c08260; *hydA2*) encoding a protein with high similarity (43%) to HydA1 (Awo_c26970) from the [FeFe]-hydrogenase of *A. woodii*. HydA2 is predicted to contain a complete H-Cluster. The genetic organization is similar to a gene cluster of *Treponema primitia* that was reported to belong to the same class as the hydrogenase-coupled formate dehydrogenase (FdhF) from *Escherichia coli*, which oxidizes formate and produces H_2_ during sugar fermentation. Based on the similarity of FdhF_Tp_ to FdhF of *E. coli* and the presence of the hydrogenase genes it was suggested for the enzyme of *T. primitia* that it directly uses molecular hydrogen as reductant [29]. A related gene cluster was also found in *Eubacterium acidaminophilum* [30]. This gene cluster consists of genes encoding hydrogenase and formate dehydrogenase subunits but the encoded hydrogenase is more complex and similar to the multimeric hydrogenase from *A. woodii*. Graentzdoerffer *et al.* [30] proposed the most simple type of a formate hydrogen lyase system.

**Figure 3:**
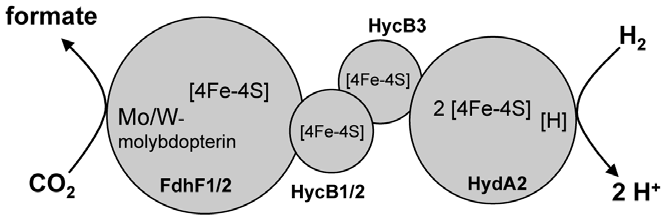
Subunit composition of the formate dehydrogenase of *A. woodii*. *fdhF1* and *fdhF2* code for sulfur and selenium containing isoenzymes, respectively. HycB1 and HycB2 may be specific for FdhF1 and FdhF2, respectively. The electron transfer subunits HycB1, HycB2 and HycB3 each have four conserved tertacysteine motifs.

Based on the analogy we suggest that Awo_c08190-Awo_c08260 encode a hydrogenase-coupled formate deyhdrogenase that oxidizes hydrogen and donates the electrons *via* the electron transfer subunits HycB3 and HycB1/2 to CO_2_ which is reduced to formate (Fig. 3).

### Re-gaining reduced ferredoxin in the carbon pathway

The methylene-THF reductase catalyzed reaction is the only considerably exergonic reaction of the pathway and, therefore, was already considered in 1977 [8] as one of the energy conserving reactions in acetogens. The methylene-THF reductase was purified from the two acetogens *Clostridium formicoaceticum* [31] and *Blautia producta* [32]. The latter one consists of a single subunit with a molecular mass of 32 kDa, contains FAD as cofactor and is NADH-dependent. In contrast, the enzyme from *C. formicoaceticum* consists of two subunits (26 and 35 kDa), has been proposed to be ferredoxin-dependent and contains FAD, FeS cluster and zinc.

In *A. woodii*, there is one gene (Awo_c09310) that encodes a soluble, 33 kDa protein with similarity to methylene-THF reductases (MetF) with a conserved flavin binding site. The preceding gene (Awo_c09300) encodes a protein with 205 amino acids and the region from amino acid 104 to 200 is similar to the C-terminal domain of methylene-THF reductases. This domain is encoded in many organisms next to *metF*, in several organisms it is even fused to *metF*. Awo_c09300 encodes for a 23 kDa protein that contains 8 conserved cysteine residues, which could coordinate two iron-sulfur clusters. Therefore it seems likely that this protein is the small subunit of the methylene-THF reductase of *A. woodii* and the entry point for electrons. Thus, the methylene-THF reductase is suggested to have a small (MetV) and large subunit (MetF) encoded by Awo_c09300 and Awo_c09310 with flavin and FeS cluster as cofactors.

**Figure 4:**
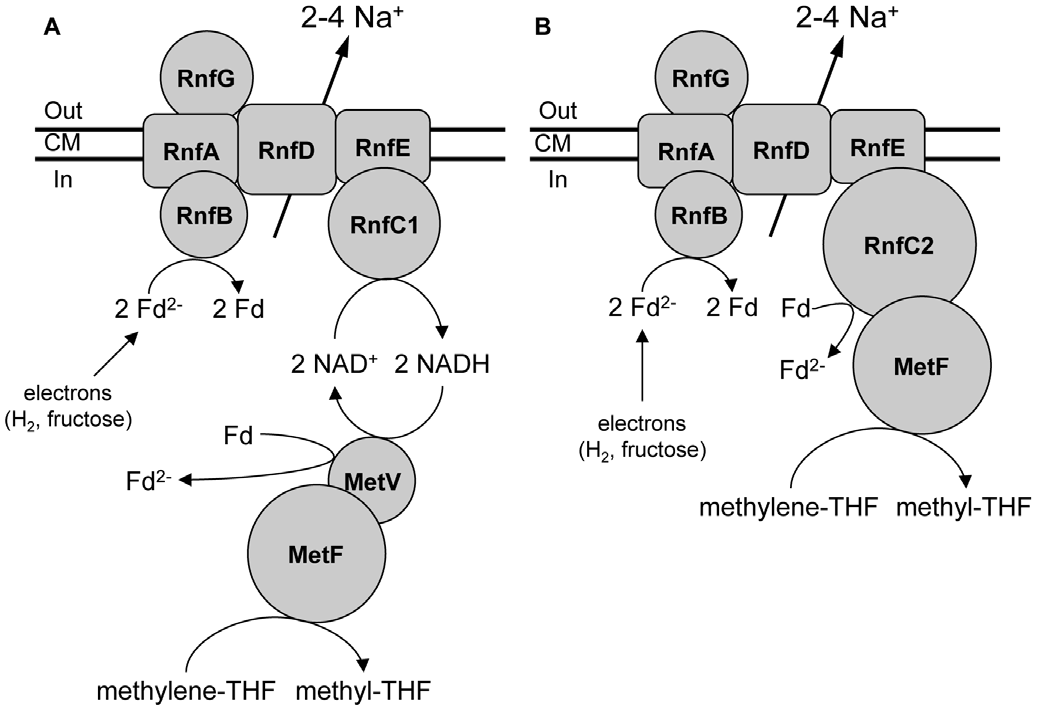
Subunit composition and model of the methylene-THF reductase of *A. woodii*. Panel A depicts an indirect coupling via NADH+H^+^ and the small subunit of the methylene-THF reductase (MetV) as electron input module, panel B a direct coupling of the methylene-THF reductase to the Rnf complex *via* RnfC2.

The reduction of methylene-THF (E_0_′ methylene-THF/methyl-THF = −200 mV) with NADH is exergonic [33] and was suggested to drive the reduction of ferredoxin with NADH [34] by electron bifurcation [26], [35]. This is supported by the predicted flavin and FeS centers (Fig. 4). Reduction of ferredoxin in this step is beneficial for the overall bioenergetics of the pathway (see below).

Interestingly, the genes encoding the small and large subunit of the methylene-THF reductase are cotranscribed with the preceding gene, Awo_c09290, indicating that it is also involved in the reduction of methylene-THF. Awo_c09290 encodes a protein with similarity (61%) to the RnfC-subunit of the Rnf complex, and the residues for binding flavin and the FeS-cluster are conserved. Thus, it is named RnfC2. In contrast to RnfC1 an extension of 220 amino acids is found at the N-terminus, which is surprisingly similar (59%) to the small subunit of the methylene-THF reductase. It is, therefore, conceivable that RnfC2 could substitute for the small subunit in the methylene-THF reductase and bind directly to the Rnf complex at the RnfC binding site. The electrons derived from the Rnf complex could then be transferred to the methylene-THF reductase without the intermediate step *via* NADH (Fig. 4, right panel). Reduced ferredoxin from the methylene-THF reductase could be reoxidized immediately at RnfB. This would fit to previous findings that this step may be membrane-associated and involved in ion translocation [36], [37].

### Energy conservation during autotrophic growth: a quantitative model

**Figure 5:**
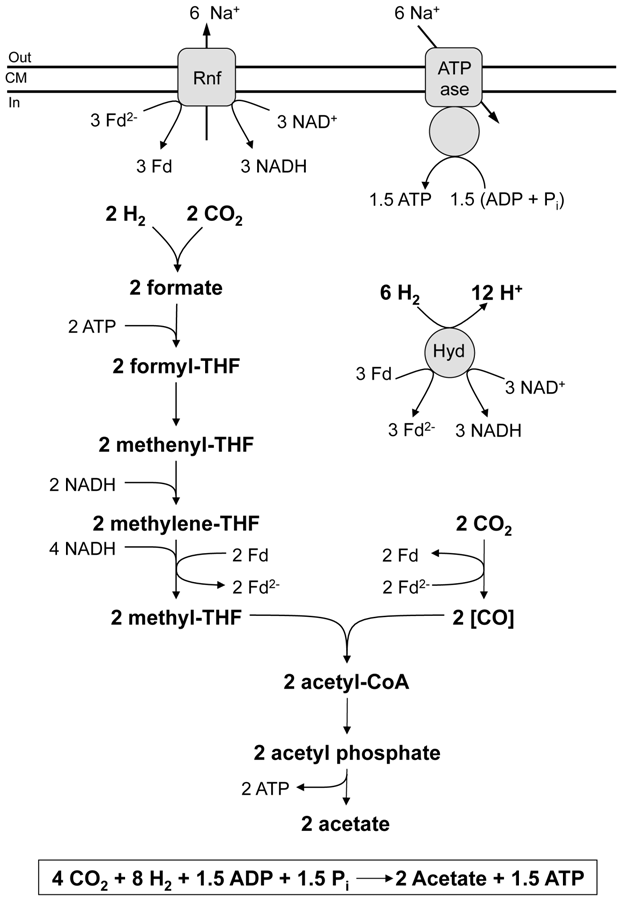
A quantitative bioenergetic model for acetogenesis from H_2_+CO_2_. The amount of ions translocated by the Rnf complex and the Na^+^ F_1_F_O_ ATP synthase are not exactly known. For sake of clarity, a Na^+^/e^−^ stochiometry of 1∶1 is assumed (based on a ΔG^O^′ value of −37 kJ/mol; E_0_′ Fd^2−/^Fd_ox_ = −500 mV; E_0_′ NADH+H^+^/NAD^+^ = −320 mV; and Δu_Na+_ (electrochemical Na^+^ potential across the cytoplasmic membrane) = −320 mV). For the ATP synthase, a Na^+^/ATP stoichiometry of 4 is assumed. The redox potentials (E_0_′) are: CO_2_/formate = −430 mV, methenyl-THF/methylene-THF = −300 mV, methylene-THF/methyl-THF = −200 mV, CO_2_/CO = −520 mV.

Based on the experimental data and the predictions from the genome sequence, a model for the bioenergetics of acetogenesis from H_2_+CO_2_ is proposed (Fig. 5). Oxidation of hydrogen is catalyzed by the electron bifurcating [FeFe]-hydrogenase, six mol of hydrogen are used to reduce three mol of NAD^+^ and three mol of ferredoxin. The three mol of ferredoxin are oxidized by the Rnf complex to reduce another three mol of NAD. Assuming a Na^+^/e^−^ stoichiometry of 1, electron transfer is accompanied by the translocation of six mol of Na^+^. Reentry of the six mol of Na^+^ allows for the production of 1.5 mol ATP by the ATP synthase, assuming a stoichiometry of 4H^+^/ATP for the ATP synthase. Two mol NADH+H^+^ are channelled into the methylene-THF dehydrogenase reaction, four mol into the methylene-THF reductase reaction. The latter may yield two mol of reduced ferredoxin by electron bifurcation that are used to reduce two mol of CO_2_ to CO. The model gives an even redox balance and postulates an ATP gain of 0.75 ATP per mol of acetate formed.

The reduction of CO_2_ to formate with H_2_ is endergonic by +3 kJ/mol under physiological standard conditions (H_2_ and CO_2_ as gases at 10^5^ Pa partial pressure, formate at 1 M concentration and pH = 7.0). As will be shown below, the threshold concentration of *A. woodii* for H_2_ at a CO_2_ partial pressure of 0.2×10^5^ Pa is 250 Pa. Under these conditions the equilibrium concentration of formate is about 0.1 mM which is in the range of the K_m_ for formate of formyl-THF synthethase, which catalyzes the second step in CO_2_ reduction to acetate, namely the formation of formyl-THF from formate, THF and ATP. The reduction of CO_2_ to formate with H_2_ in *A. woodii* is therefore most likely not energy dependent. Consistent with this hypothesis, the reduction of CO_2_ to formate with H_2_ in *A. woodii* is catalyzed by a cytoplasmic enzyme complex composed of a [FeFe]-hydrogenase, two electron transfer iron-sulfur proteins and a molybdenum/tungsten-dependent formate dehydrogenase.

The free energy change associated with 2 CO_2_ reduction with 4 H_2_ to acetate under physiological conditions (CO_2_ partial pressure of 0.2×10^5^ Pa, a H_2_ partial pressure of 250 Pa and an acetate concentration of 10 mM at pH 7) is about −40 kJ/mol which is sufficient to drive the phosphorylation of only less than 1 mol ADP considering that under the irreversible conditions in cells between 50 and 80 kJ/mol are required for the synthesis of 1 mol ATP [8].

### The hydrogen threshold

**Figure 6:**
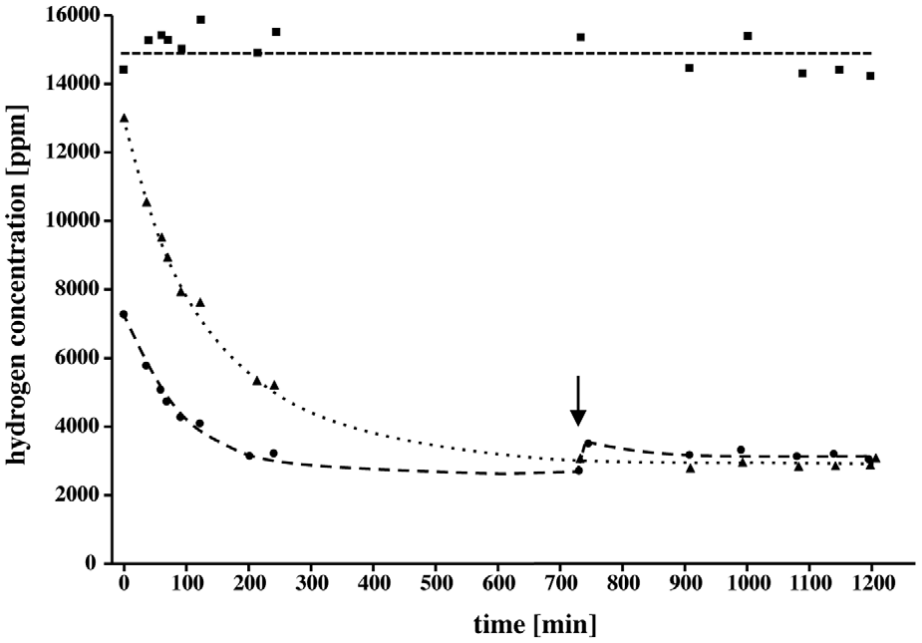
The hydrogen threshold concentration for acetogenesis from H_2_+CO_2_. Time course of hydrogen oxidation by cell supensions of *A. woodii* with CO_2_ as terminal electron acceptor. Assays received 1.5 (▴) or 1 ml (•) hydrogen. One assay did not contain cells (▪). At the indicated time point, hydrogen was added again to proof the viability of the cells.

To determine the minimal hydrogen concentration that can be used by *A. woodii* cell suspensions were incubated under different hydrogen partial pressures and the hydrogen consumption was monitored by gas chromatography. *A. woodii* was able to oxidize hydrogen down to 2500 ppm (250 Pa) which leads to a redox potential of about −340 mV (Fig. 6). Therefore, we can conclude that the minimal hydrogen concentration in the ecosystem must be above 250 Pa to allow litho-autotrophic growth on H_2_+CO_2_. Acetogenesis from H_2_+CO_2_ has a free energy change of −95 kJ per mol which leads to a theoretical hydrogen threshold (ΔG = 0 kJ/mol) of about 10 Pa. If we assume an ATP gain of n ATP/acetate this leads to the equation:
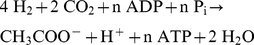
(3)With a ΔG of −45 kJ/mol (ΔG for ADP phosphorylation ∼50 kJ/mol) and n = 0.5 the theoretical threshold for H_2_ increases to approximately 100 Pa. For n = 1 the threshold is reached at approximately 1100 Pa. The measured and theoretical hydrogen concentrations are in very good accord leading to an ATP gain in acetogenesis of 0.5 to 1 mol ATP/mol acetate. It has to be considered that the measured threshold is always lower than the theoretical one due to a partial uncoupling of metabolism and energy conservation. The threshold for H_2_ of 250 Pa is very high compared to the thresholds of methanogens without cytochromes which is one order of magnitude lower [38]. Therefore *A. woodii* can not compete with those methanogens in its natural habitat for H_2_+CO_2_ as carbon and energy source.

### Conclusions

The acetogenic bacterium *A. woodii* is a prime candidate for an organism that catalyzes what may be one of the first life sustaining processes on earth based on CO_2_ fixation. Acetate formation from hydrogen and carbon dioxide by the Wood-Ljungdahl pathway is catalyzed by cytoplasmic, soluble enzymes. Energy conservation is *via* a chemiosmotic mechanism of the simplest type with just two enzyme complexes: the ATP synthase and the Rnf complex. The Rnf complex couples oxidation of reduced ferredoxin with reduction of NAD^+^ and concomittant export of Na^+^ from the cells. The use of Na^+^ as coupling ion underlines current views that a sodium ion-based bioenergetics evolutionary predates a proton-based bioenergetics [39]. The energy barriers that have to be overcome in the pathways of electron and carbon flow are not driven by a membrane-bound, reverse electron flow but by soluble enzymes that use electron bifurcation. Moreover, even the little potential drop between NADH (E_0_′ = −320 mV) and methylene-THF (E_0_′ = −200 mV) may be used bioenergetically, to reduce ferredoxin. *A. woodii* lives on the thermodynamic edge of life and uses a fascinating repertoire of enzymes to cope with energy limitations. The metabolism is optimized to (i) get as much as possible reduced ferredoxin to fuel the Rnf complex and (ii) not to use the sodium motive force to overcome energy barriers but the soluble electron bifurcating [FeFe]-hydrogenase and methylene-THF reductase and a hydrogen-coupled formate dehydrogenase. Altogether, this allows for the synthesis of about to 0.5–1 mol ATP per mol of acetate produced.

## Materials and Methods

### Growth of *A. woodii*

*A. woodi* was cultivated at 30°C. The medium was prepared as described [40]. Fructose was used as carbon source at a final concentration of 20 mM. Growth was followed by measuring the optical density at 600 nm (OD_600_).

### Sequencing strategy

Whole-genome sequencing was performed using the 454 FLX pryrosequencing system (Roche 454, Branford, USA). Total DNA of *A. woodii* was extracted by MasterPure™ complete DNA purification kit (Epicentre, Madison, USA). The isolated DNA was used to create 454-shotgun libraries following the GS FLX general library protocol (Roche 454, Branford, USA). One medium lane of a Titanium picotiter plate was used for sequencing of the library, resulting in 232121 shot gun reads. The reads were *de novo* assembled using the Roche Newbler assembly software 2.3 (Roche 454).

### Gene prediction and annotation

Annotation was done by using the ERGO tool from Integrated Genomics with a two-step approach. Initially, all proteins were screened against Swiss-Prot and NCBI databases and available protein sequences from other completed genomes by using FASTA3. All predictions were verified and modified manually by comparing the protein sequences with Pfam, GenBank, ProDom, COG, and Prosite public databases. All coding sequences were searched for similarities to protein families and domains using CD-search [41].

### Proteome analysis

*A. woodii* was grown either on fructose or on hydrogen and carbon dioxide. Cells were harvested during early exponential, late exponential and stationary growth. 2D gel electrophoresis (500 µg soluble protein, pH 4–7) was done according to [42]. Staining and analysis of the gels and identification of proteins by mass spectrometry were done as described earlier [43] except that a Proteome Analyzer 4800 was used and the signal-to-noise ratio of the TOF-TOF measurement was raised to 10. An *A. woodii* data base was used for searching the peak lists.

### Transcriptional organization

Cells were grown on fructose and harvested in the exponentiell growth phase. Isolation of RNA and analysis of transcriptional organization was performed as described previously [44].

### Determination of the H_2_ threshold concentration

H_2_-threshold concentrations were determined with 1 g cells (wet mass) harvested in the exponential growth phase and resuspended in 1 ml medium. The 2 ml cell suspensions were transferred to 150 ml H_2_-free Müller–Krempel flasks (Bülach, Germany) sealed by Perbunan rubber stoppers (Deutsch & Naumann, Berlin, Germany) and repeatedly evacuated and filled with 80% N_2_/20% CO_2_ to remove remaining traces of H_2_. Then H_2_ was added up to 2000 Pa. The flasks were then rapidly shaken at 30°C. Gas samples were taken with a gas-tight syringe (Hamilton, Bonaduz, Switzerland) at first every 10 min and then later every hour. After the H_2_ concentration remained constant, H_2_ was added again to ascertain that the same H_2_ threshold was reached a second time. The samples were analyzed by gas chromatography using a stainless steel separation column (0.5 nm molecular sieve: 80/100 mesh; length = 2 m×4 mm) kept at 120°C. H_2_ was detected with a thermal conductivity detector (GC-8A; Shimadzu) (carrier gas, N_2_; flow rate, 40 mL/min) [45]. The peak heights/areas were proportional to the H_2_ concentration. Calibrations were done with a calibration gas mixture (1,000 ppmv) from Messer Industriegase, Sulzbach, Germany.

## Supporting Information

Figure S1 **The soluble proteome of** ***A. woodii*** **grown either on fructose (greenimage) or on H_2_+CO_2_ (redimage).** The dual channel image was created with the Delta 2D software (Decodon GmbH, Greifswald, Germany). Proteins were prepared during early exponential growth, separated in a pH gradient 4–7 and stained with colloidal Coomassie Brillant Blue.
(TIF)

Table S1 **Differentially regulated proteins during growth on fructose** ***versus*** **growth on hydrogen and carbon dioxide.**
(DOC)
