# Supplementary figures and images for: An Ancient Pathway Combining Carbon Dioxide Fixation with the Generation and Utilization of a Sodium Ion Gradient for ATP Synthesis
Source: PLoS One. 2012 Mar 29;7(3):e33439. doi: 10.1371/journal.pone.0033439 (PMC3315566; doi:10.1371/journal.pone.0033439)

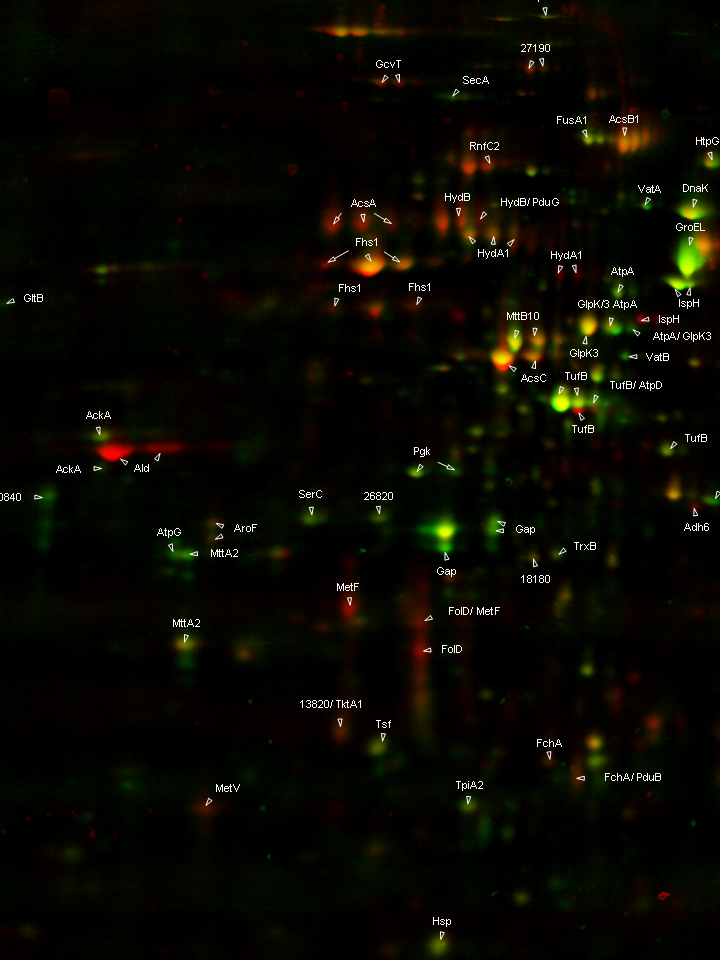

Supplement: Figure S1 — The soluble proteome of A. woodii grown either on fructose (greenimage) or on H2+CO2 (redimage). The dual channel image was created with the Delta 2D software (Decodon GmbH, Greifswald, Germany). Proteins were prepared during early exponential growth, separated in a pH gradient 4–7 and stained with colloidal Coomassie Brillant Blue. (TIF) [file pone.0033439.s001.tif]
