# Supplementary material for: An Ancient Pathway Combining Carbon Dioxide Fixation with the Generation and Utilization of a Sodium Ion Gradient for ATP Synthesis
Source: PLoS One. 2012 Mar 29;7(3):e33439. doi: 10.1371/journal.pone.0033439 (PMC3315566; doi:10.1371/journal.pone.0033439)
Supplement: Table S1 — Differentially regulated proteins during growth on fructose versus growth on hydrogen and carbon dioxide. (DOC) [file pone.0033439.s002.doc]

Tab. S1: Differentially regulated proteins during growth on fructose *versus* growth on hydrogen and carbon dioxide.

| ratio H_2_ + CO_2_ early exp _vs_ fructose early exp 1 | ratio H_2_ + CO_2_ early exp _vs_ fructose early exp 2 | label | locus tag |
| --- | --- | --- | --- |
| 14.82 | 9.98 | HydA1 5 | Awo_c26970 |
| 12.96 | 5.73 | Ald | Awo_c24350 |
| 8.69 | 6.47 | HydA1 4 | Awo_c26970 |
| 4.83 | 3.69 | Ald 2 | Awo_c24350 |
| 4.49 | 1.74 | FolD | Awo_c09280 |
| 3.58 | 0.64 | Fhs1 | Awo_c09260 |
| 2.62 | 1.72 | AcsE | Awo_c10730 |
| 2.62 | 0.20 | Fhs1 5 | Awo_c09260 |
| 2.58 | 2.91 | IspH | Awo_c09760 |
| 2.29 | 2.88 | AcsA | Awo_c10740 |
| 2.27 | 1.78 | AcsE 2 | Awo_c10730 |
| 2.14 | 2.02 | HydB | Awo_c26980 |
| 2.11 | 1.51 | 13820/ TktA1 | Awo_c13820/ Awo_c03710 |
| 2.09 | 2.93 | GcvT | Awo_c32800 |
| 2.05 | 1.54 | FolD/ MetF | Awo_c09280/ Awo_c09310 |
| 2.04 | 2.90 | HydB/ PduG | Awo_c26980/ Awo_c25860 |
| 2.02 | 2.06 | 27190 | Awo_c27190 |
| 2.01 | 2.56 | RnfC2 | Awo_c09290 |
| 1.96 | 0.75 | GcvT 2 | Awo_c32800 |
| 1.92 | 2.39 | Adh6 | Awo_c25410 |
| 1.75 | 1.53 | Fhs1 2 | Awo_c09260 |
| 1.73 | 1.89 | AcsC | Awo_c10720 |
| 1.68 | 2.18 | AcsA 3 | Awo_c10740 |
| 1.65 | 1.53 | AcsD | Awo_c10710 |
| 1.64 | 2.35 | AcsA 2 | Awo_c10740 |
| 1.64 | 1.84 | AcsB2 | Awo_c33680 |
| 1.61 | 2.04 | HydA1 3 | Awo_c26970 |
| 1.58 | 1.05 | AcsC 1 | Awo_c10720 |
| 1.50 | 1.73 | Fhs1 3 | Awo_c09260 |
| 1.35 | 1.68 | HydA1 | Awo_c26970 |
| 1.31 | 1.00 | Fhs1 4 | Awo_c09260 |
| 0.57 | 0.59 | IspH 2 | Awo_c09760 |
| 0.55 | 0.73 | TpiA2 | Awo_c24510 |
| 0.54 | 0.32 | PduE | Awo_c25870 |
| 0.53 | 0.07 | IspH 3 | Awo_c09760 |
| 0.53 | 0.60 | RfbB1/ VatI fr | Awo_c02320/ Awo_c23950 |
| 0.52 | 0.58 | TufB/ AtpD | Awo_c09160/ Awo_c02230 |
| 0.49 | 0.61 | GlpK3/ AtpA | Awo_c12770/ Awo_c02210 |
| 0.47 | 0.67 | Pgk 2 | Awo_c24520 |
| 0.47 | 0.68 | DnaK | Awo_c26630 |
| 0.45 | 0.26 | 26820 | Awo_c26820 |
| 0.43 | 0.72 | AtpA | Awo_c02210 |
| 0.43 | 0.56 | Pgk | Awo_c24520 |
| 0.42 | 0.38 | GroEL | Awo_c16490 |
| 0.41 | 0.71 | 07580 | Awo_c07580 |
| 0.40 | 0.57 | SerC | Awo_c28180 |
| 0.39 | 0.65 | AtpA/ GlpK3 | Awo_c02210/ Awo_c12770 |
| 0.38 | 0.54 | FtsZ | Awo_c09640 |
| 0.34 | 0.42 | Gap 2 | Awo_c24530 |
| 0.29 | 0.39 | MttA2 | Awo_c07520 |
| 0.27 | 0.48 | SecA | Awo_c28060 |
| 0.27 | 0.37 | VatA | Awo_c23910 |
| 0.27 | 0.38 | VatB | Awo_c23900 |
| 0.27 | 0.43 | GltB | Awo_c07370 |
| 0.26 | 0.40 | AtpG | Awo_c02220 |
| 0.25 | 0.21 | 30840 | Awo_c30840 |
| 0.22 | 0.27 | Gap | Awo_c24530 |

Data are derived from Fig. S1 and quantification was done with the Delta 2D software (Decodon GmbH, Greifswald, Germany). Some proteins have multiple forms in the SDS page and anre numbered according to Fig. S1.
